# Viable Bacteria Associated with Red Blood Cells and Plasma in Freshly Drawn Blood Donations

**DOI:** 10.1371/journal.pone.0120826

**Published:** 2015-03-09

**Authors:** Christian Damgaard, Karin Magnussen, Christian Enevold, Martin Nilsson, Tim Tolker-Nielsen, Palle Holmstrup, Claus Henrik Nielsen

**Affiliations:** 1 Section for Periodontology, Microbiology and Community Dentistry, Department of Odontology, Faculty of Health and Medical Sciences, University of Copenhagen, Copenhagen, Denmark; 2 Institute for Inflammation Research, Department of Infectious Diseases and Rheumatology, Rigshospitalet, Copenhagen University Hospital, Copenhagen, Denmark; 3 Department of Clinical Immunology and Blood Centre, Rigshospitalet, Copenhagen University Hospital, Hvidovre, Denmark; 4 Costerton Biofilm Center, Department of International Health, Immunology and Microbiology, Faculty of Health and Medical Sciences, University of Copenhagen, Copenhagen, Denmark; FDA, UNITED STATES

## Abstract

**Objectives:**

Infection remains a leading cause of post-transfusion mortality and morbidity. Bacterial contamination is, however, detected in less than 0.1% of blood units tested. The aim of the study was to identify viable bacteria in standard blood-pack units, with particular focus on bacteria from the oral cavity, and to determine the distribution of bacteria revealed in plasma and in the red blood cell (RBC)-fraction.

**Design:**

Cross-sectional study. Blood were separated into plasma and RBC-suspensions, which were incubated anaerobically or aerobically for 7 days on trypticase soy blood agar (TSA) or blue lactose plates. For identification colony PCR was performed using primers targeting 16S rDNA.

**Setting:**

Blood donors attending Capital Region Blood Bank, Copenhagen University Hospital, Rigshospitalet, Hvidovre, Denmark, October 29^th^ to December 10^th^ 2013.

**Participants:**

60 donors (≥50 years old), self-reported medically healthy.

**Results:**

Bacterial growth was observed on plates inoculated with plasma or RBCs from 62% of the blood donations. Growth was evident in 21 (35%) of 60 RBC-fractions and in 32 (53%) of 60 plasma-fractions versus 8 of 60 negative controls (p = 0.005 and p = 2.6x10^-6^, respectively). *Propionibacterium acnes* was found in 23% of the donations, and *Staphylococcus epidermidis* in 38%. The majority of bacteria identified in the present study were either facultative anaerobic (59.5%) or anaerobic (27.8%) species, which are not likely to be detected during current routine screening.

**Conclusions:**

Viable bacteria are present in blood from donors self-reported as medically healthy, indicating that conventional test systems employed by blood banks insufficiently detect bacteria in plasma. Further investigation is needed to determine whether routine testing for anaerobic bacteria and testing of RBC-fractions for adherent bacteria should be recommended.

## Introduction

In general, the risk of mortality and morbidity following blood transfusion is low, and has declined over recent years [1–3]. Infection resulting from the introduction of a pathogen into a person through blood transfusion are known as transfusion-transmitted infections (TTIs) [3], and such infections remains a leading cause of post-transfusion mortality and morbidity [4–5].

A recent meta-analysis of 18 randomized trials showed remarkably high rates of nosocomial-infections: 16.9% following a liberal transfusion strategy (hemoglobin threshold for transfusion ranging from 9.0 to 11.3 g/dL), and 11.8% following a more restrictive strategy (hemoglobin threshold ranging from 6.4 to 9.7 g/dL) [6]. The odds ratio for developing infectious complications following RBC transfusion has been estimated to 1.88 [7]. In clear contrast, bacterial growth is usually found in less than 0.1% of blood units using conventional test systems, such as BacT/ALERT [8–9], which is applied to 89.5% of all platelet apheresis performed in USA in 2011 [10]. There is currently no data to explain the discrepancy between the high rates of post-transfusional infections and low rates of bacterial contamination in the available literature.

Infectious complications to blood transfusion include sepsis, pneumonia, abscesses, wound infection, meningitis, hemolysis, empyema, urine tract infection and fever [11]. Such infections may be partly accounted for by an inhibitory effect of the transfusion *per se* on the immune system [12–14]. However, another cause might be unrecognized bacterial contamination of the transfused blood units.

Bacteria in donor blood may derive from unidentified infections in the donor, or contamination during venipuncture. Previous studies have shown that daily activities such as chewing, tooth brushing, and flossing facilitate translocation of bacteria into the blood stream [15–18]. In particular, the common inflammatory disease periodontitis, affecting more than 50% of the population older than 50 years, causes breakdown of tooth supporting tissues as well as deepening and ulceration of periodontal pockets through which bacteria may gain access to the blood stream [15, 18–20]. However, periodontitis is currently not an exclusion criterion for blood donation. Notably, the indigenous microbiota of the periodontal pockets includes commensals of the skin [21].

While conventional tests for bacterial contamination of donor blood are based on sampling from the thrombocyte-fraction [22], sampling from plasma or thrombocytes does not reveal bacteria adhering to red blood cells (RBCs), which may constitute a reservoir of blood-borne bacteria [23]. Thus, opsonization of bacteria by complement enables bacteria to adhere to RBCs via complement receptor 1 (CR1), a phenomenon referred to as immune adherence [23–25].

We hypothesized that the high frequency of post-transfusional infections is due to unrevealed contamination of donor blood, including the RBC fraction that is not routinely subjected to screening. The aim of the study was therefore 1) to identify viable bacteria in standard blood-pack units, with particular focus on bacteria from the oral cavity, and 2) to determine the distribution of bacteria revealed in plasma and in the RBC-fraction.

## Methods

### Sample size

The present study is cross-sectional. Sample size was estimated using a two-sided power analysis with μ(0) = 40, μ(1) = 10, Σ = 50, α = 0.005 and a power of 95%. The total sample size required for the study was 56. The final sample size was, however, adjusted to a total of 60 participants, allowing a rate of 7.5% of eligible subjects, who could withdraw their consent.

### Ethics

The study and consent procedure were approved by The Ethics Committee for The Capital Region of Denmark (#H-4–2012–020). All donors attended the Capital Region Blood Bank, Copenhagen University Hospital, Rigshospitalet, Hvidovre, Denmark, October 29^th^ to December 10^th^ 2013. All donors gave informed written consent prior to blood donation.

### Blood specimen collection

Blood was drawn from 60 donors self-reported as medically healthy (age 50 years or older) from the antecubital vein after topical disinfection with a combination of 2% chlorhexidine gluconate and 70% isopropyl alcohol for 30 seconds, followed by 30 seconds drying time, in accordance with WHO guidelines [26–28]. The first 30 mL blood drawn was collected into a pre-sample bag to minimize the risk of contamination from insertion of the needle. The following 450 mL of blood was drawn into triple blood-pack units containing citrate phosphate dextrose solution (CPD) (#R6488; Fenwal^TM^, Mont Saint Guibert, Belgium). The tube connecting the needle with the pre-sample bag and the 450 mL triple blood-pack units was welded off. The triple blood-pack units were then stored at room temperature until fractionation and culturing (within 9 hours). The pre-sample bag was discarded.

### Blood fractionation

In a laminar flow hood at Institute for Inflammation Research, Department of Infectious Diseases and Rheumatology, Rigshospitalet, Copenhagen University Hospital, Copenhagen, Denmark, the bottom hose on the blood-pack unit was disinfected twice with 85% alcohol and afterwards cut with a sterile scissor. The first 30 ml blood was discarded to minimize risk of contamination from cutting of the hose. The following 30 ml were poured directly into two sterile 15 mL tubes, which were then fractioned under sterile conditions into plasma and blood cells by centrifugation at 400 x *g*. The RBC-fraction was washed twice in sterile phosphate buffered saline (PBS).

### Isolation of viable bacteria from blood

0.5 mL of plasma and 0.5 mL washed RBC-suspension were plated out separately under sterile conditions on trypticase soy blood agar (TSA) plates, containing 5 mg/L hemin and 50 μg/L vitamin K, and incubated at 37°C under anaerobic conditions in the presence of 10% CO_2_, 10% H_2_, and 80% N_2_, or aerobically in the presence of 5% CO_2_. Another 0.5 mL of each fraction was handled similarly and incubated on blue lactose plates under aerobic conditions. All plates were incubated at Department of Clinical Microbiology, Rigshospitalet, Copenhagen, Denmark, for seven days at 37°C.

As negative controls, TSA plates and blue lactose plates were incubated either non-inoculated or with CPD solution, collected in the same manner as blood from blood-pack units, and subsequently diluted 1:3 in sterile PBS.

### Detection of colony forming units

All plates were examined for colonies after 7 days of incubation. If positive, the number of colonies was counted and the plate was photographed. Colonies were then individually transferred to fresh plates to obtain monocultures for identification of species. The re-plated colonies were incubated for 4 days.

### Colony PCR and 16S rDNA sequence analysis

For identification of bacteria, colony PCR was performed using primers targeting the bacterial 16S rDNA, as described by Bosshard et al. 2004 [29]. Colony PCR was performed at Costerton Biofilm Center, Department of International Health, Immunology and Microbiology, Faculty of Health and Medical Sciences, University of Copenhagen, Copenhagen, Denmark. Bacterial 16S rDNA isolate sequences were compared with taxon sequences in the Human Oral Microbiome Database (HOMD), the NCBI database, and the Ribosomal Database Project.

### Statistical methods

Using Fishers exact test, bacterial growth observed on plates with plasma, RBCs or the negative control CPD, was compared. McNemar test (paired) was used to evaluate differences in number of plates with bacterial growth. Mann-Whitney test was used to examine differences between the number of colony forming units on plates with RBC- and plasma fractions, versus growth-positive controls.

## Results

**Fig 1:**
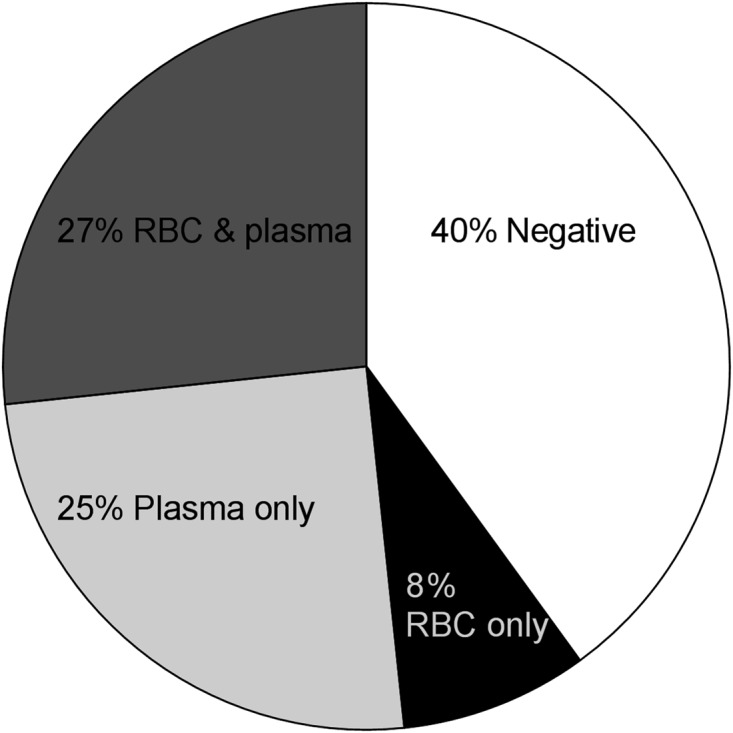
Frequency of viable bacteria in donor blood. Freshly drawn blood from 60 healthy blood donors was fractioned into plasma and RBCs and plated on trypticase soy blood agar plates under aerobic or anaerobic conditions, and on blue lactose plates under aerobic conditions. RBC- or plasma-fractions were defined as positive if at least 1 colony was observed on at least one of the six plates. Shown are the frequencies of donors for whom bacteria were found in the RBC-fraction only, in the plasma-fraction only, in both fractions, or in none of the fractions.

Bacterial growth, recorded on basis of at least 1 colony, was observed on plates inoculated with plasma and/or RBCs from 37 (62%) of the 60 blood donations (Fig. 1), compared to a frequency of 5% of the negative control samples (Fishers exact test: p = 1.4×10^-11^).

By blood fraction, growth was evident in 21 (35%) of 60 RBC-fractions and in 32 (53%) of 60 plasma-fractions versus 8 (13%) of 60 negative controls (Fisher’s exact test: p = 0.005 and p = 2.6×10^-6^, respectively). RBC- and plasma-fractions did not differ significantly with respect to the number of plates with bacterial growth (McNemar test: p = 0.39). Notably, in five cases (14% of the growth-positive blood units), bacteria were detected in the RBC-fraction only.

The number of colony forming units (CFU) was 2.0 and 2.3 on growth-positive plates derived from RBC- and plasma-fractions, respectively, versus 0.5 on growth-positive control plates (Mann-Whitney: p = 0.002 and p = 1.2×10^-6^, respectively).

As shown in Table 1, the species isolated included *Propionibacterium acnes* (in 23% of the donations), *Staphylococcus epidermidis* (in 38%), *Staphylococcus caprae* (in 8%), *Micrococcus luteus* (in 5%), and *Acinetobacter lwoffii* (in 3%).

**Table 1:** Bacterial species identified^1^.

	RBC	Plasma	CPD in PBS
*Acinetobacter lwoffii* ^2^	2	1	
*Aerococcus viridans* ^2^	1		
*Bacillus thuringiensis*		1	
*Bacillus pumilus*		1	
*Bacillus stratosphericus*	1		
*Brachybacterium sp*.		1	
*Dietzia papillomatosis* ^2^		1	
*Granulicatella sp*. ^2^	1		
*Micrococcus luteus* ^2^	2	1	2
*Moraxella osloensis* ^2^		1	
*Planobacterium taklimakanense*	1		
*Prolinoborus fasciculus*	1		
*Propionibacterium acnes* ^2^	9	8	
*Propionibacterium avidum* ^2^		2	
*Propionibacterium granulosum* ^2^		1	
*Pseudomonas mosselii* ^2^	2		
*Sphingomonas aurantiaca*		1	
*Sphingomonas yabuuchiae*			1
*Staphylococcus aureus* ^2^		1	
*Staphylococcus caprae* ^2^	4	2	
*Staphylococcus epidermidis* ^2^	8	19	2
*Staphylococcus saprophyticus subsp*. *saprophyticus*	3	2	
*Staphylococcus warneri* ^2^		1	
Not determined	2	2	3

^1^ Bacteria were identified by comparing the 16S rDNA sequence from the isolate with taxons from HOMD, the NCBI database, and the Ribosomal Project Database. All species were identified with minimum 98.5% confidence. RBC: Red blood cell fraction. CPD: Citrate phosphate dextrose solution (anticoagulant containing negative control). PBS: Phosphate buffered saline buffer.

Growth of the given species in *n* out of 60 blood donations is shown.

^2^ Bacteria previously found associated with nosocomial-infections.

## Discussion

Post-transfusion infection is known to occur at a higher rate [4–7] than the low freuency of positive findings in conventional bacterial screening systems based on pH-testing [20], detection of CO_2_ [8, 10], and swirling of platelet concentrates [9]. While platelet concentrates are routinely tested under aerobic conditions only [6], we tested the plasma and RBC fractions of donor blood for content of viable bacteria, by cultivation on TSA plates and blue lactose plates under both aerobic and anaerobic conditions. Our method uses direct culturing from both RBCs and plasma, which enables detection of viable bacteria contained in both blood compartments.

Growth in more than one of the media incubated was, however, derived from a surprisingly high proportion of blood units (52.8%), indicating that the findings were not false-positive contaminants [30]. The species most frequently found were *Staphylococci* spp., *Propionibacterium* spp., *Bacillus* spp. and *Micrococcus* spp.

Severeal previous studies have focussed on identification of bacteria in patient blood or transfused blood components, once patients developed clinical symptoms of TTIs [1–3, 5]. Thus, the BACTHEM study included patients with transfusion-related adverse events, such as fever, chills, drop in blood pressure, shock, isolated dyspnea, malaise, anxiety and digestive distress [2]. In direct blood agar culture 77% were posive for bacteria [2], and the bacteria found in the blood components were Gram-negative rods in 46% of cases, Gram-positive cocci in 28% and Gram-positive rods in 21% [2]. The bacteria isolated in our study were primarily Gram-positive cocci and Gram-positive rods, and the most preponderant species were also fund in the BACTHEM study, e.g. *Staphylococcus epidermidis* and *Propionibacterium acnes*. The second-most abundant bacteria in the BACTHEM study was the Gram-negative *Acinetobacter lwoffii*, which accounted for only 3% in the present study. Since *Acinetobacter lwoffii* has been isolated from the forearm of up to 48% of healthy donors [2], it seems propable that *Acinetobacter lwoffii*, like *Staphylococcus epidermidis* may have been introduced into blood specimens at the collecting stage, although the first 30 mL of collected blood were discarded. It should be noted, however, that e.g. *Staphylococcus epidermidis*, *Propionibacterium acnes*, *Micrococcus luteus*, *Acinetobacter lwoffii* and *Staphylococcus aureus* also inhabit the periodontium, and may have been present in the blood stream of donors [15–18]. Unlike the BACTHEM study, we did not find the Gram-negative rods *Klebsiella*, *Escherichia coli*, *Serratia*, *Enterobacter*, *Yersinia* and *Proteus*. Studies based on recognized TTIs are likely to underestimate the frequency of bacteremia for a number of reasons: i) Studies are often based on standard methods for bacterial screening only, ii) if none of the transfused blood components are available, patients are excluded from the studies, iii) patients may be receiving antimicrobials at the time of blood culture, iv) participation may be non-uniform and often voluntary, and v) fever and other symptoms may not be interpreted as caused by bacteria, and vi) bacteremia is presumably often asymptomatic in immunocompetent individuals.

Like the present study, other investigations have been based on sterility testing of randomly selected blood products. However, Soeterboek et al. reported considerably less contamination, i.e. 1% positive RBC products, and 0.5% of the total blood products tested, than suggested by our data [31]. Their findings were, however, based on BacT/ALERT-testing only. In accordance with our data, however, the most frequently isolated bacteria were *Staphylococcus epidermidis*, followed by *Propionibacterium acnes* and other propionibacteria [31]. Kunishima et al. reported even less frequent contamination of blood products, i.e. 0.18%, all of which were RBC concentrates, *Propionibacterium acnes* being the most frequent contaminant [32]. Their findings were, however, based on cultivation in bottles of thioglycollate and soybean casein digest broth media, rather than the direct cultures employed here.

The majority of the bacterial species identified has been associated with nosocomial infections like sepsis, endocarditis, pneumonia, meningitis, urinary tract and wound infections [30, 33–36]. Notably, TTI with *Staphylococcus epideremidis* has been reported to cause potemtially fatal sepsis [37–39].

Interestingly, *Pseudomonas mosselii* [40], *Granulicatella sp*., [41] and *Aerococcus viridans* [42, 43], all capable of causing endocarditis, were found solely in samples of RBC from three donors.

At least three factors may contribute to the high frequency of contaminated blood products found in this study: Unlike most other studies, we only included donors of 50 years of age, or older. This inclusion criterion increases the risk of unreported infections such as periodontitis, which might explain the high prevalence of bacterial growth detected. Secondly, the majority of bacteria identified in the present study were either facultative anaerobic (59.5%) or anaerobic (27.8%) species, which are not likely to be detected using current screening procedures. Thirdly, the RBC fraction is not routinely tested for contamination.

Obviously, larger studies are required to confirm our findings, and since symptomatic TTIs are rare, screening of RBC preparations and cultivation under anaerobic conditions are probably not recommendable in general. Such procedures may be applied to blood products intended for immunocompromised individuals, e.g. patients undergoing chemotherapy. Moreover, surveillance for staphylococci and propionibacteria in patients with post-transfusion infections may be considered. Finally, it should be tested specifically whether periodontal disease may enhance the frequency of donor blood contamination.
